# Riboswitch-mediated inducible expression of an astaxanthin biosynthetic operon in plastids

**DOI:** 10.1093/plphys/kiab428

**Published:** 2021-09-07

**Authors:** Shreya Agrawal, Daniel Karcher, Stephanie Ruf, Alexander Erban, Alexander P Hertle, Joachim Kopka, Ralph Bock

**Affiliations:** Max-Planck-Institut für Molekulare Pflanzenphysiologie, Am Mühlenberg 1, D-14476 Potsdam-Golm, Germany

## Abstract

The high-value carotenoid astaxanthin (3,3′-dihydroxy-β,β-carotene-4,4′-dione) is one of the most potent antioxidants in nature. In addition to its large-scale use in fish farming, the pigment has applications as a food supplement and an active ingredient in cosmetics and in pharmaceuticals for the treatment of diseases linked to reactive oxygen species. The biochemical pathway for astaxanthin synthesis has been introduced into seed plants, which do not naturally synthesize this pigment, by nuclear and plastid engineering. The highest accumulation rates have been achieved in transplastomic plants, but massive production of astaxanthin has resulted in severe growth retardation. What limits astaxanthin accumulation levels and what causes the mutant phenotype is unknown. Here, we addressed these questions by making astaxanthin synthesis in tobacco (*Nicotiana tabacum*) plastids inducible by a synthetic riboswitch. We show that, already in the uninduced state, astaxanthin accumulates to similarly high levels as in transplastomic plants expressing the pathway constitutively. Importantly, the inducible plants displayed wild-type–like growth properties and riboswitch induction resulted in a further increase in astaxanthin accumulation. Our data suggest that the mutant phenotype associated with constitutive astaxanthin synthesis is due to massive metabolite turnover, and indicate that astaxanthin accumulation is limited by the sequestration capacity of the plastid.

## Teaser

Inducible expression of a synthetic astaxanthin operon in plastids alleviates the growth phenotype of constitutive pathway expression and provides insights into carotenoid biosynthesis bottlenecks.

## Introduction

Astaxanthin (3,3′-dihydroxy-β,β-carotene-4,4′-dione) is a ketocarotenoid (oxidized carotenoid) synthesized by some marine bacteria (e.g. *Agrobacterium aurantiacum*) and algae (e.g. *Hematococcus pluvialis*), and the red yeast *Xanthophyllomyces dendrorhous*. Astaxanthin is one of the most powerful antioxidants produced in nature (Giuliano et al., 2000; Ralley et al., 2004) and, therefore, has attracted wide attention for its pharmacological activities, including its potential to prevent cancer and aging-related diseases (Jia et al., 2020). In addition, the compound is in great demand by the food and cosmetic industries, and is widely used as a dietary supplement and colorant. By far the largest part of the astaxanthin produced is used in aquaculture, where the pigment is an expensive component of fish feed and required to confer the coloration of salmon and trout flesh (Nogueira et al., 2017). Since commercial-scale production of astaxanthin in microorganisms is very costly, engineering of the astaxanthin pathway into crop plants has been pursued to provide a cheaper source of the pigment (Ralley et al., 2004; Gerjets and Sandmann, 2006; Hasunuma et al., 2008; Zhu et al., 2008; Harada et al., 2014; Lu et al., 2017; Nogueira et al., 2017). Transplastomic approaches, in which the pathway genes are expressed from the plastid (chloroplast) genome, have been particularly successful in that very high levels of astaxanthin accumulation (of up to 1% of the plant dry weight) have been attained (Hasunuma et al., 2008; Lu et al., 2017; Fuentes et al., 2018).

When engineered into plants, astaxanthin synthesis utilizes the natural carotenoid β-carotene as substrate which in turn is derived from the methylerythritol 4-phosphate (MEP) pathway of isoprenoid biosynthesis (Enfissi et al., 2005; Misawa, 2011). Conversion of β-carotene to astaxanthin requires introduction of hydroxyl and keto groups at the 3,3′ and 4,4′ positions, respectively, of the two β-ionone rings (Ralley et al., 2004; Gerjets and Sandmann, 2006; Tao et al., 2006; Lu et al., 2017). The β-carotene ketolase and hydroxylase genes from *Brevundimonas* species have successfully been used to introduce these enzymatic activities into various seed plants (Hasunuma et al., 2008; Campbell et al., 2015; Farré et al., 2016; Mortimer et al., 2016; Mortimer et al., 2017).

In previous research, we have demonstrated that the strong constitutive expression of the astaxanthin biosynthetic pathway in transgenic plastids results in depletion of endogenous carotenoid species and nearly complete replacement of the carotenoids that are naturally present in the photosynthetic apparatus by astaxanthin (Liguori et al., 2017; Lu et al., 2017; von Oort et al., 2018; Xu et al., 2020). While being capable of photoautotrophic growth, the transplastomic plants displayed growth retardation and delayed development (Lu et al., 2017). Whether impaired plant growth is due to inefficiency of photosynthetic light harvesting and electron transport in the absence of natural carotenoids (Liguori et al., 2017; von Oort et al., 2018; Xu et al., 2020), or rather caused by metabolic drain and/or depletion of isoprenoid precursors, is currently not known.

To gain insights into the bottlenecks involved in astaxanthin synthesis and accumulation in seed plants, and determine the cause of the growth phenotype of transplastomic plants that constitutively produce astaxanthin to high levels, here we used a previously designed inducible transgene expression system for plastids to control the flux into astaxanthin synthesis by application of a chemical inducer. We report that application of this system to a synthetic astaxanthin operon in tobacco (*Nicotiana tabacum*) plastids largely alleviates the growth phenotype associated with pathway expression from a constitutive promoter. Comprehensive characterization of isoprenoid metabolism in transplastomic plants provides insights into ketocarotenoid synthesis, stability, and turnover, and suggests carotenoid sequestration as the key factor limiting astaxanthin accumulation in plants.

## Results

### Introduction of an inducible astaxanthin biosynthetic operon into the tobacco plastid DNA

**Figure 1:**
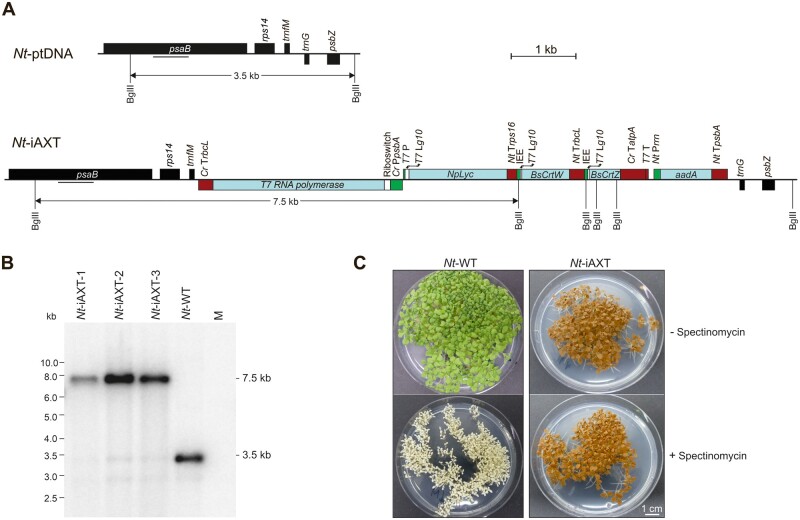
Generation of homoplasmic transplastomic tobacco plants for riboswitch-inducible expression of a synthetic astaxanthin operon. A, Physical maps of the targeting region in the tobacco plastid genome (ptDNA; upper panel) and the modified region in transplastomic *Nt*-iAXT lines harboring the synthetic astaxanthin operon and the elements of the RAmpER system (Verhounig et al., 2010; Emadpour et al., 2015). The recognition sites of restriction endonucleases used for RFLP analysis and the resulting fragment sizes are indicated. The binding sites of the hybridization probe for RFLP analysis are represented as black horizontal bars. *Cr* P*psbA*: chloroplast *psbA* promoter from *Chlamydomonas reinhardtii*; *Nt* P*rrn*: plastid rRNA operon promoter from *N. tabacum*; *T7* P: T7 RNA polymerase promoter from bacteriophage T7; *Cr* T*rbcL*: 3′ UTR of *rbcL* from *C. reinhardtii*; *Nt* T*psbA*: 3′ UTR of *psbA* from *N. tabacum*; *Cr* T*atpA*: 3′ UTR of *atpA* from *C. reinhardtii*; *Nt* T*rbcL*: 3′ UTR of *rbcL* from *N. tabacum*; *Nt* T*rps16*: 3′ UTR of *rps16* from *N. tabacum*; *T7* L*g10*: 5′ UTR of *gene10* from phage T7; IEE (Zhou et al., 2007; Legen et al., 2018). B, RFLP analysis of transplastomic tobacco plants generated with the iAXT construct for RAmpER-dependent inducible expression of the synthetic astaxanthin operon. Total DNA was digested with the restriction enzyme BglII, and fragments were detected by hybridization with a radiolabeled *psaB*-specific probe (cf. panel A). *Nt*-WT: wild-type tobacco; M: molecular weight marker. C, Seed assays to confirm homoplasmy of transplastomic plants. Wild-type (*Nt*-Wt) seeds and T1 seeds from an *Nt*-iAXT plant were germinated on synthetic medium in the presence or absence of spectinomycin. Absence of antibiotic-sensitive progeny and absence of green seedlings indicate the homoplasmic state of the transplastomic line with respect to the presence of both the *aadA* gene and the astaxanthin operon. + Spectinomycin: 500 mg L^−1^ spectinomycin in the culture medium; − Spectinomycin: control with no antibiotic in the culture medium.

In previous research, we constructed an inducible expression system for plastids. The system, dubbed RNA amplification-enhanced riboswitch (RAmpER), relies on expression of an RNA polymerase gene (derived from phage T7) from the plastid genome that is regulated at the translational level by a synthetic theophylline-responsive riboswitch (Verhounig et al., 2010; Emadpour et al., 2015). Expression of the transgene of interest is controlled at the transcriptional level by a promoter recognized specifically by the T7 RNA polymerase (T7RNAP). In the presence of the inducer molecule of the riboswitch, theophylline, T7RNAP is synthesized at a relatively low level (Verhounig et al., 2010) which is sufficient to initiate strong transcription of the transgene (Emadpour et al., 2015). When applied to control the synthesis of a toxic protein (the HIV antigen Nef; Zhou et al., 2008; Marusic et al., 2009) in plastids, the RAmpER system completely prevented the development of the mutant phenotype conferred by constitutive protein expression (Emadpour et al., 2015), presumably by allowing chloroplast development and thylakoid biogenesis to proceed in the absence of toxic levels of the recombinant protein. To test if RAmpER also alleviates the negative consequences of transplastomic metabolic pathway expression, we replaced the constitutive promoter previously used to drive expression of a synthetic astaxanthin operon in plastids (Lu et al., 2017) by the T7RNAP promoter and additionally integrated a T7RNAP transgene under riboswitch control into the plastid genome of tobacco plants (Figure 1A). The astaxanthin biosynthetic operon comprises the lycopene β-cyclase gene from daffodil (*Narcissus pseudonarcissus*), *NpLyc* (to stimulate synthesis of β-carotene, the precursor of ketocarotenoids; Apel and Bock, 2009), and the β-carotene ketolase and hydroxylase genes from *Brevundimonas sp.* strain SD212, *BsCrtW and BsCrtZ* (Figure 1A; Lu et al., 2017).

The resulting transformation construct was introduced into tobacco plastids by particle gun-mediated (biolistic) transformation (Svab and Maliga, 1993; Bock, 2015). Plastid transformation experiments and selection for spectinomycin resistance conferred by the chimeric *aadA* marker gene (encoding aminoglycoside 3″-adenylyltransferase) resulted in the isolation of several independent transplastomic events (subsequently referred to as *Nt*-iAXT lines, for inducible astaxanthin-synthesizing *N. tabacum* plants), three of which were further characterized. To eliminate residual copies of the (highly polyploidy) untransformed plastid genome (Greiner et al., 2020), additional rounds of plant regeneration under selective conditions were conducted prior to assessment of the transplastomic status by restriction fragment length polymorphism (RFLP) analysis via Southern blotting (Figure 1B). These assays revealed the expected 3.5 kb BglII restriction fragment in the wild type, and the expected 7.5 kb fragment in all transplastomic lines (Figure 1, A and B). Virtual absence of the wild-type-size fragment from the transplastomic lines provided preliminary evidence of homoplasmy for the transformed plastid genome. Homoplasmy was ultimately confirmed by inheritance assays that revealed a genetically and phenotypically homogeneous T1 progeny (Figure 1C), consistent with uniparentally maternal plastid inheritance (Greiner et al., 2015).

### Growth and development of transplastomic *Nt*-iAXT plants

**Figure 2:**
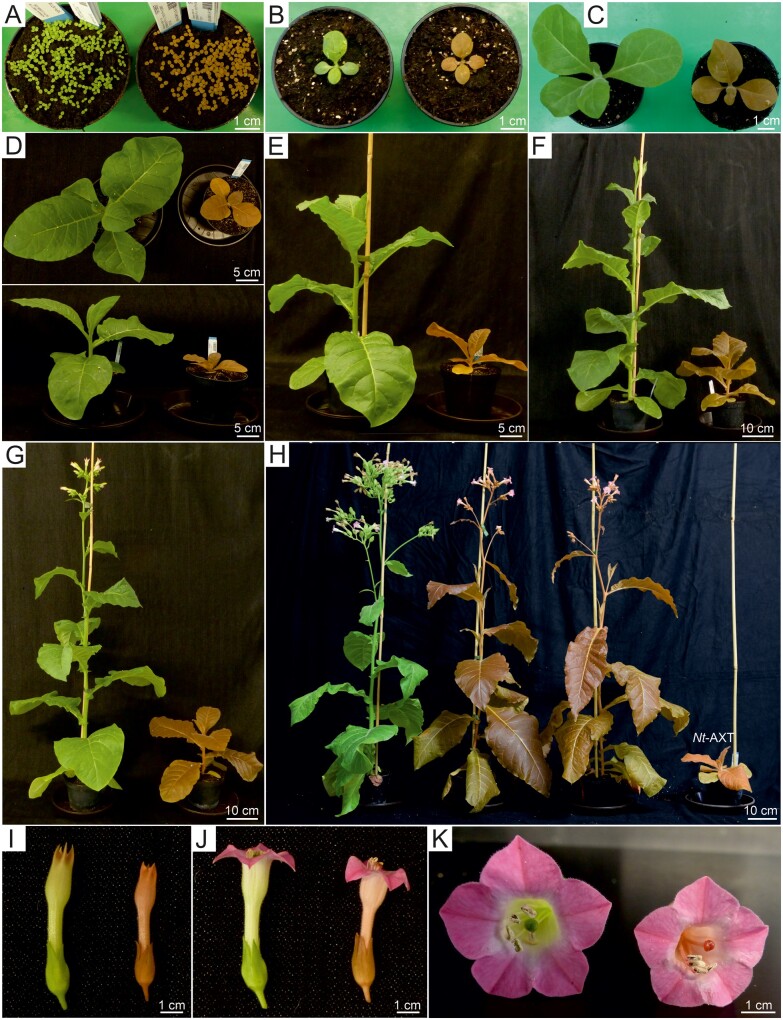
Phenotype of transplastomic *Nt*-iAXT plants in comparison to wild-type tobacco plants. A–H, Plants photographed at different stages of growth; transplastomic *Nt*-iAXT plants are shown on the right (orange-brown phenotype), and wild-type tobacco plants are shown on the left (green). A, 10 d, (B) 17 d, (C) 24 d, (D) 31 d, (E) 38 d, (F) 48 d, (G) 52 d, and (H) 70 d after sowing. In panel (H), a transplastomic plant constitutively expressing the astaxanthin pathway (*Nt*-AXT; Lu et al., 2017) is included for comparison. I–K, Flower phenotype of *Nt*-iAXT plants. I, Side view of transplastomic (right) and wild-type (left) flowers shortly before opening of the corolla. J, Flowers after opening of the corolla. K, Top view showing the red stigma and style of *Nt*-iAXT flowers.

Transplastomic *Nt*-iAXT plants displayed a conspicuous orange–brown phenotype both upon in vitro culture (Figure 1C) and when grown in soil under standard greenhouse conditions (Figure 2). This striking leaf coloration is reminiscent of the phenotypes of transgenic and transplastomic plants that constitutively express astaxanthin biosynthetic enzymes (Hasunuma et al., 2008; Lu et al., 2017; Mortimer et al., 2017). Remarkably, the *Nt*-iAXT plants developed their conspicuous pigmentation phenotype already in the absence of riboswitch induction with theophylline. This finding suggests that the inherent leakiness of the RAmpER system (Emadpour et al., 2015) is sufficient to produce the pathway enzymes to amounts that confer substantial flux into astaxanthin biosynthesis. The RAmpER leakiness may be an inherent property of the riboswitch and/or due to the presence of a bacteriophage-type RNA polymerase in plastids (referred to as nucleus-encoded plastid RNA polymerase, NEP) that has overlapping promoter specificity (Liere and Maliga, 1999; Magee and Kavanagh, 2002; Emadpour et al., 2015).

When growth and development of the transplastomic *Nt*-iAXT plants was followed over the entire life cycle, a slight growth delay relative to wild-type plants was observed (Figure 2). The onset of flowering occurred ∼2 weeks later in *Nt*-iAXT plants (Figure 2, G and H). However, compared to transplastomic plants expressing the astaxanthin operon constitutively (referred to as *Nt*-AXT; Lu et al., 2017), plant growth was greatly improved (Figure 2H). Similar to *Nt*-AXT plants (Lu et al., 2017), pigmentation of the inducible plants was strikingly altered also in non-leafy tissues, including all floral organs (Figure 2, I–K).

**Figure 3:**
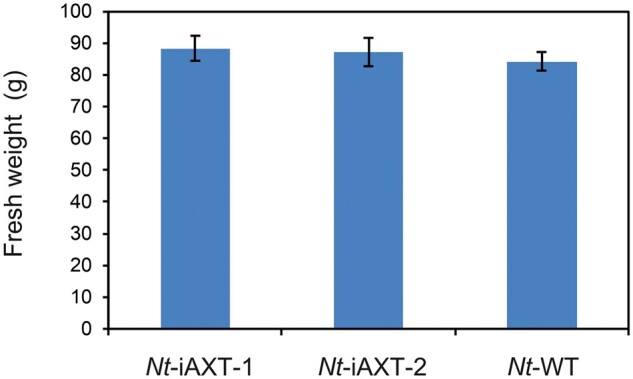
Comparison of leaf biomass of wild-type plants (*Nt*-WT) and two independently generated transplastomic (*Nt*-iAXT) tobacco lines. Samples were collected from plants at the 12-leaf stage, and the total fresh weight of all leaves of a given plant was determined. Error bars indicate the standard deviation (*n* = 6).

One of the attractive features of tobacco in molecular farming is the high biomass production per unit area and time (Knuckles et al., 1979; Tusé et al., 2014; Fuentes et al., 2016). To assess whether our metabolic manipulation has an effect on the overall yield of leaf tissue, we measured the biomass of transplastomic plants and wild-type plants at the onset of flowering. At this stage, wild-type plants and *Nt*-iAXT plants both had 12 leaves. When their total fresh weight was determined, no difference in the overall biomass yield between transplastomic and wild-type plants was observed (Figure 3), indicating that, with the exception of the slight developmental delay (Figure 2), expression of the inducible astaxanthin operon does not entail a yield penalty.

### Transcript accumulation from the synthetic astaxanthin operon upon riboswitch induction with theophylline

**Figure 4:**
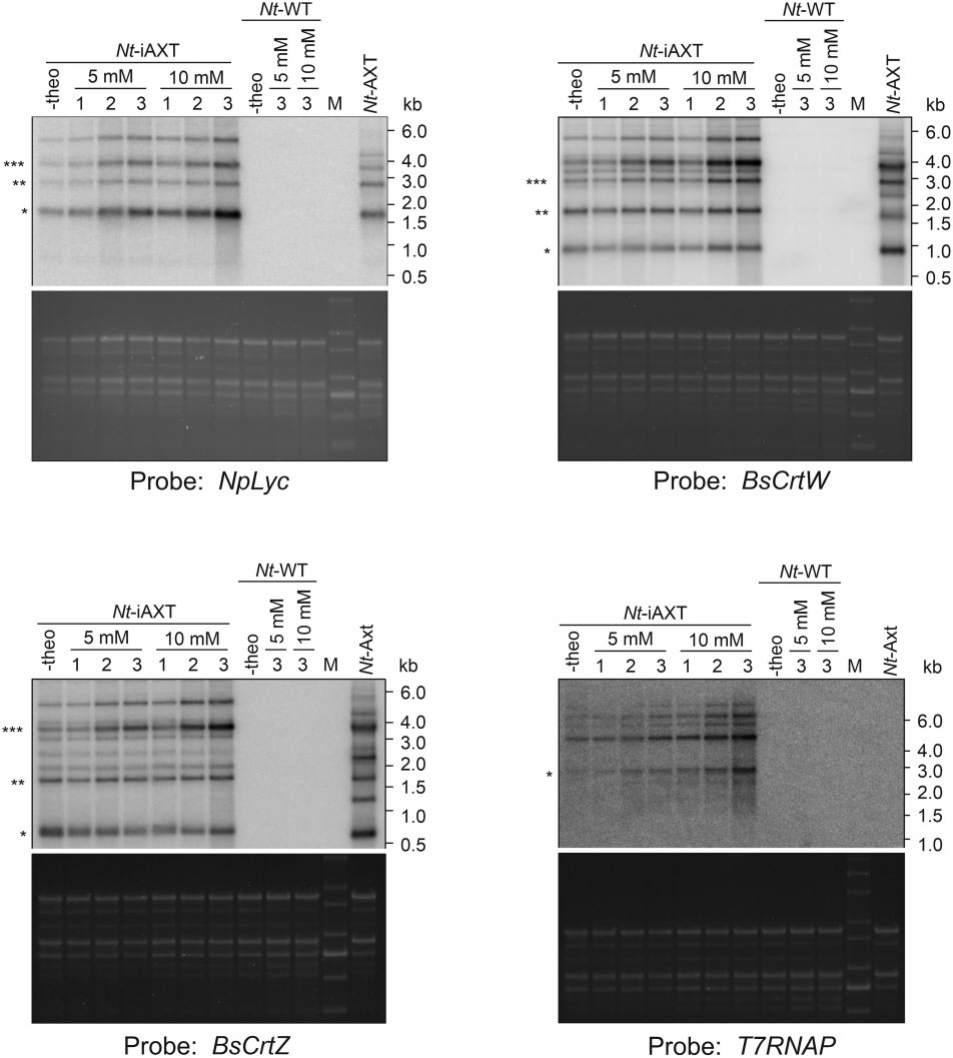
Northern blot analyses to examine mRNA accumulation in *Nt*-iAXT transplastomic plants and assess the induction of operon expression in response to theophylline application. mRNA accumulation was analyzed in leaf number 5 of 6-week-old plants after watering with 5 or 10 mM theophylline over a period of 3 d. Plants were watered daily and the numbers on top of the lanes indicate the number of waterings with theophylline solution the plants received. Asterisks indicate the expected transcript sizes for *monocistronic, **dicistronic, and ***tricistronic mRNAs derived from the astaxanthin operon. Additional minor hybridizing transcript species were not characterized. Larger transcript species are likely the result of read-through transcription due to inefficient transcription termination in plastids (Zhou et al., 2007; Lu et al., 2013). The ethidium bromide-stained gel prior to blotting is shown as a control for equal loading below each blot. M, RNA size marker; -theo, control plants without theophylline application; *Nt*-WT, wild type; *Nt*-AXT, transplastomic plant constitutively expressing the astaxanthin pathway (Lu et al., 2017).

Next, we analyzed the accumulation of mRNAs of the astaxanthin biosynthetic operon in response to theophylline induction of the riboswitch. To this end, the RAmpER system was induced by watering with 5 mM or 10 mM theophylline solution, and mRNA accumulation for the three operon genes was followed over three consecutive days by northern blot analysis (Figure 4). For both theophylline concentrations, a clear time-dependent induction of transcript accumulation was seen. As expected, the response was also dose-dependent in that watering with 10 mM theophylline triggered a stronger induction than watering with 5 mM. Theophylline concentrations >15 mM turned out to be toxic to the plants, as evidenced by the appearance of necrotic spots on the leaves. When *Nt*-iAXT plants were fully induced with 10 mM theophylline, mRNA accumulation for all three operon genes reached similar or even higher levels than in *Nt*-AXT plants that express the operon constitutively (Figure 4).

The strongest increase in accumulation of monocistronic mRNAs was observed for *NpLyc*, the first gene in the operon. The two downstream cistrons, *BsCrtW and BsCrtZ*, showed only a moderate induction of the fully processed monocistronic mRNA, but strong induction of the tricistronic operon transcript (Figure 4). This finding suggests that the efficiency of intercistronic processing limits accumulation of the monocistronic *BsCrtW and BsCrtZ* mRNAs. The intercistronic expression element (IEE; Zhou et al., 2007) separating the three operon genes is known to be the target of an RNA-binding protein (High Chlorophyll Fluorescent 107, HCF107), whose levels may become limiting, when its binding sequence is strongly overexpressed (Legen et al., 2018). Thus, depletion of HCF107 may result in inefficient intercistronic processing and/or reduced protection of monocistronic mRNAs from exoribonucleolytic degradation (Pfalz et al., 2009; Legen et al., 2018).

Interestingly, we also observed an increase in *T7RNAP* transcript abundance upon RAmpER induction with theophylline (Figure 4). This was unexpected, because the synthetic riboswitch is a translational switch. However, coverage of the mRNA with ribosomes during active translation can enhance transcript stability (Caroca et al., 2013; Zoschke and Bock, 2018), thus potentially explaining the increased *T7RNAP* mRNA accumulation upon translation activation by the riboswitch.

### Astaxanthin accumulation increases upon RAmpER induction

We next wanted to determine if riboswitch induction of operon expression results in increased accumulation of astaxanthin. To this end, *Nt*-iAXT plants at the six-leaf stage were watered with theophylline as described above, and samples were collected from leaf number 2 and leaf number 5 (counted from the bottom of the plant) after induction for 1, 2, or 3 d.

**Figure 5:**
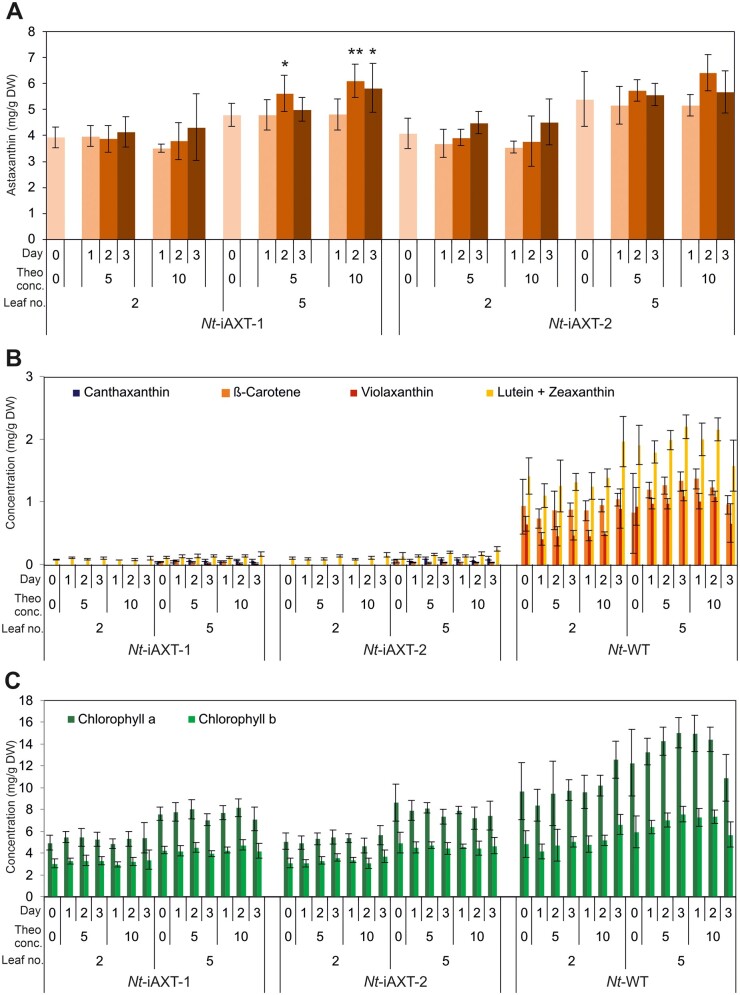
Time course analysis of pigment accumulation upon induction with 5 mM or 10 mM theophylline for 3 d. A, Astaxanthin content. B, Carotenoid contents. C, Chlorophyll contents. Plants were induced at the six-leaf stage by watering with theophylline solution (once per day for three consecutive days). DW, leaf dry weight; Day, number of times the plants were watered with theophylline solution (once per day); Conc., concentration of the theophylline (Theo) solution used for induction; WT, wild type. Error bars represent the sd (*n* = 6). Significant changes in astaxanthin accumulation on induction compared to the uninduced state are marked by asterisks (Student’s *t* test; *P < 0.05; **P < 0.01). Note that the increase in astaxanthin contents in line *Nt*-iAXT-2 is slightly above the significance criteria (*P* = 0.078 at 10 mM theophylline after 2 d).

Significant increases in astaxanthin accumulation were obtained on induction with 10 mM theophylline for 2 and 3 d in leaf number 5 (Figure 5A). The stronger effect on leaf 5 is likely due to leaf 5 being younger (and, unlike leaf 2, not yet fully expanded) and, therefore, having a higher metabolic activity. To further test this hypothesis, induction experiments were also performed with older plants (at the 12-leaf stage), and samples were collected from leaf number 8. Only minor differences in astaxanthin accumulation were observed (Supplemental Figure S1), in line with the idea that low metabolic activity in mature and old leaves prevents efficient induction of astaxanthin synthesis. No significant changes in accumulation of other carotenoids or chlorophylls were observed upon RAmpER induction of the astaxanthin biosynthetic operon (Figure 5, B and C).

### Chloroplasts in inducible transplastomic lines have a fully developed thylakoid network

**Figure 6:**
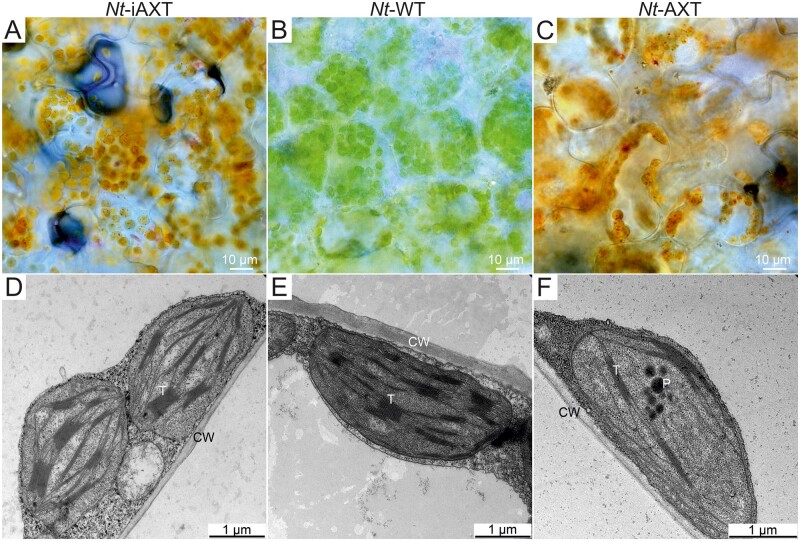
Microscopic images of leaf mesophyll cells from wild-type (*Nt*-WT) and transplastomic tobacco plants expressing the astaxanthin biosynthetic operon either inducibly (*Nt*-iAXT) or constitutively (*Nt*-AXT). A–C, Light microscopic images. Dark red particles within the chloroplasts of transplastomic leaves represent sites of aggregation and/or crystallization of astaxanthin (Lu et al., 2017). D–F, TEM images. Note that, while the transplastomic plants engineered to produce astaxanthin constitutively (Lu et al., 2017) show an underdeveloped thylakoid network (T) and accumulation of large plastoglobules (P), the inducible transplastomic plants display normally developed thylakoid stacks and are similar to wild-type chloroplasts. CW, cell wall.

In contrast to the transplastomic lines expressing the astaxanthin operon constitutively, the *Nt*-iAXT plants displayed greatly improved growth properties and also showed darker leaf pigmentation (Figure 2H), tentatively suggesting that their photosynthetic apparatus is less impaired (and their chlorophyll contents are higher) than in *Nt*-AXT plants. To test this hypothesis, comparative microscopic investigations of chloroplasts were undertaken (Figure 6).

Light microscopic analysis revealed the presence of big red particles inside chloroplasts of both *Nt*-iAXT plants (Figure 6A) and *Nt*-AXT plants (Figure 6C; Lu et al., 2017). As suggested previously, these red particles likely represent aggregates or crystals of astaxanthin (Lu et al., 2017). When chloroplast ultrastructure was investigated by transmission electron microscopy (TEM), striking differences were seen between *Nt*-iAXT and *Nt*-AXT plants (Figure 6, D–E). While *Nt*-AXT chloroplasts had poorly developed thylakoids and displayed massive accumulation of lipophilic material in large osmophilic, plastoglobule-like particles (Figure 6F), *Nt*-iAXT chloroplasts showed a fully developed thylakoid system that was virtually indistinguishable from that of wild-type chloroplasts (Figure 6, D and E). Consistent with faithful lipid incorporation into thylakoid membranes, *Nt*-iAXT plants also lacked the accumulation of aberrant lipophilic particles seen in *Nt*-AXT chloroplasts (Figure 6, D and F).

### Astaxanthin accumulates to comparable levels in *Nt*-AXT and *Nt*-iAXT plants

**Figure 7:**
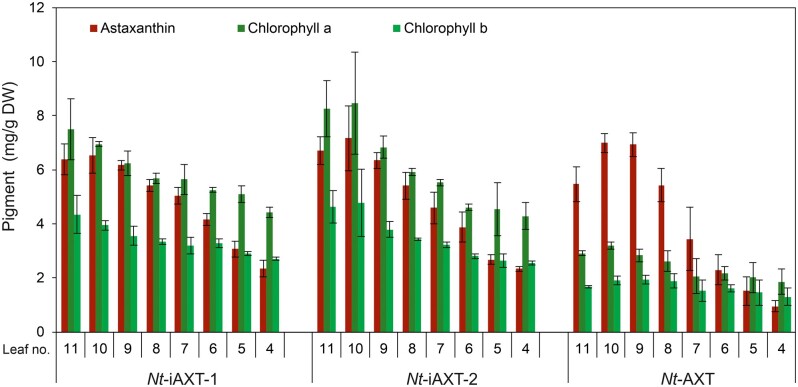
Comparison of astaxanthin and chlorophyll accumulation in transplastomic plants engineered for RAmpER-dependent expression of the synthetic astaxanthin operon (*Nt*-iAXT) and those engineered to express the operon constitutively (*Nt*-AXT; Lu et al., 2017). The diagram shows a developmental series of eight consecutive leaves (leaves number 4–11 from plants at the 12-leaf stage; leaves numbered from the bottom). Note that, although the astaxanthin content is similar in the two transplastomic lines, the chlorophyll content in *Nt*-iAXT is nearly twice as high as in *Nt*-AXT. Error bars represent the sd (*n* = 3). DW, dry weight.

Astaxanthin contents in *Nt*-AXT plants were previously determined in plants grown in sterile culture on synthetic sucrose-containing medium (Lu et al., 2017). To be able to directly compare astaxanthin accumulation in constitutive and inducible transplastomic lines under photoautotrophic conditions, *Nt*-AXT and *Nt*-iAXT plants were grown on soil under standard greenhouse conditions and in the absence of the chemical inducer theophylline. To determine the impact of leaf age on astaxanthin levels, a developmental series of leaves was harvested and subjected to pigment analysis. Interestingly, despite the strong differences in plant growth and chloroplast ultrastructure (Figures 2H and 6, D–F), *Nt*-iAXT plants accumulated astaxanthin to comparable levels as *Nt*-AXT plants (Figure 7). By contrast, the chlorophyll content of *Nt*-iAXT plants was more than 2-fold higher than that of *Nt*-AXT plants. This finding is consistent with the visual pigmentation phenotype (Figure 2H), the observed differences in thylakoid development (Figure 6, D–F), and a large body of previous work that had established that thylakoid biogenesis and chlorophyll accumulation are highly coordinated (Wang and Grimm, 2015; Armarego-Marriott et al., 2019).

### Detection of carotenoid degradation products and depletion of reactive oxygen species-scavenging compounds in astaxanthin synthesizing plants

The very similar astaxanthin accumulation levels in *Nt*-AXT and *Nt*-iAXT plants raise the intriguing question, what causes the severe growth retardation of the transplastomic plants that express the pathway constitutively. A possible explanation could be that the constitutive lines synthesize more astaxanthin than the inducible lines, but suffer from high astaxanthin turnover. This could be due to the carotenoid storage capacity of the plastid limiting astaxanthin accumulation. The relatively small increase in astaxanthin accumulation achievable by theophylline induction (Figure 5) lends circumstantial support to this idea and may suggest that the astaxanthin levels attained in *Nt*-AXT and *Nt*-iAXT plants (Figure 7) are close to the upper limit possible. This interpretation would be consistent with previous work that had suggested an important role of carotenoid sequestration by lipids and carotenoid-binding proteins in the control of carotenoid levels in algal and plant plastids (Rabbani et al., 1998; Li et al., 2012; Kilambi et al., 2013; Nogueira et al., 2013). Thus, degradation of excess astaxanthin in the transplastomic plants expressing the pathway constitutively, conceivably, could result in a futile cycle of synthesis and degradation, and at the same time, depletion of precursors from the isoprenoid pathway. The enzymatic degradation of carotenoids in plants is catalyzed by a family of carotenoid cleavage dioxygenases (CCDs; Aider et al., 2012; Gonzalez-Jorge et al., 2013; Moreno et al., 2021), while non-enzymatic breakdown is largely triggered by reactive oxygen species (ROS; Havaux, 2013).

**Figure 8:**
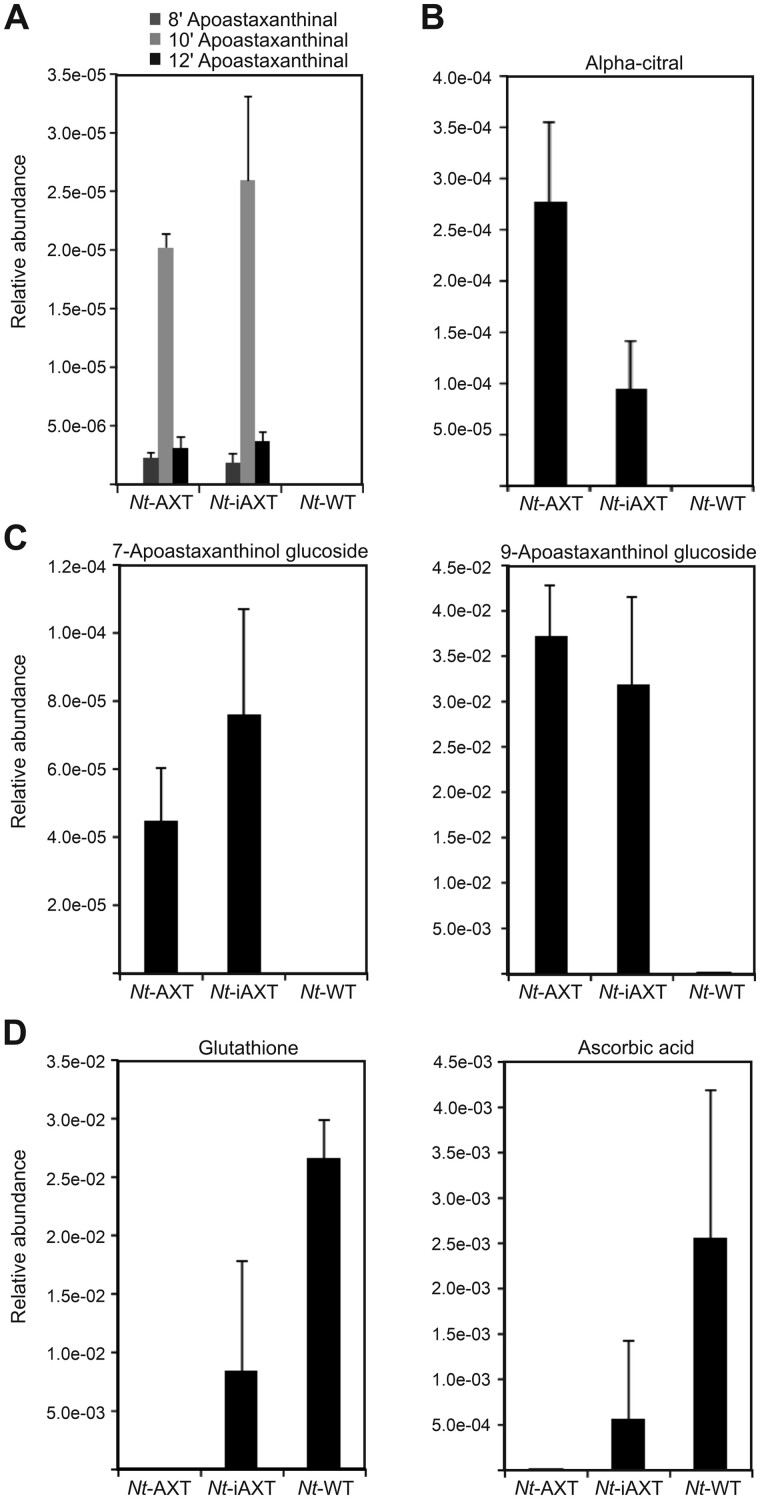
Quantification of selected metabolites by LC/GC-MS analysis. A, Long-chain oxidation products of astaxanthin. B, α-citral, a volatile isoprenoid, specifically accumulating in astaxanthin-synthesizing plants. C, Short-chain oxidation products of astaxanthin. D, Glutathione and ascorbate, two polar metabolites that act as antioxidants. Error bars represent the sd (*Nt*-iAXT: *n* = 6, *Nt*-AXT: *n* = 3, *Nt*-WT: *n* = 3). See also Supplemental Dataset S1.

To test whether the immediate breakdown products of astaxanthin accumulate to different levels in *Nt*-AXT and *Nt*-iAXT plants, LC-MS analysis was undertaken. These measurements revealed that the long-chain oxidation products of astaxanthin, 8′-apoastaxanthinal, 10′-apoastaxanthinal, and 12′-apoastaxanthinal, accumulated to similar levels in constitutive and inducible lines (Figure 8A). In both lines, 10′-apoastaxanthinal was more abundant than 8′- and 12′-apoastaxanthinal. All three compounds were also detected upon non-enzymatic oxidative degradation of astaxanthin (i.e. reaction of pure astaxanthin with atmospheric oxygen in the dark at 55°C; Etoh et al., 2012). However, other auto-oxidation products, including the short-chain counterparts of the observed long-chain products as well as 7-apoastaxanthinal, 9-apoastaxanthinone, 11-apoastaxanthinal, and the main auto-oxidation product 13-apoastaxanthinone (Etoh et al., 2012), were not detected in our comprehensive multiplexed LC-MS assays. Since these breakdown products are expected to be present in the volatile fraction, volatile metabolites were analyzed by headspace SPME-GC-MS profiling.

In the volatile leaf emissions, 7- and 9-apocarotenoids were detected in both *Nt*-AXT and *Nt*-iAXT plants, including α-citral (geranial; Figure 8B), β-cyclocitral, and geranylacetone, as well as β-ionone, α-ionone, and β-ionon-5,6-epoxide (Supplemental Dataset S1). However, the non-enzymatic astaxanthin breakdown products 7-apoastaxanthinal and 9-apoastaxanthinone were not found. The non-cyclic apocarotenoid α-citral, which was reported to act as a microtubule-targeting cytotoxin in plants (Chaimovitsh et al., 2010), accumulated to higher levels in *Nt*-AXT plants than in *Nt*-iAXT plants (Figure 8B). Other oxidation and dehydration products of lipophilic metabolites accumulated to similar levels (e.g. fatty acid-derived nonenal, nonadienal and pentadecanal, and phytol-derived neophytadiene), whereas methylsalicylate accumulated only in the constitutive lines (Supplemental Dataset S1).

To unravel the fate of the expected short-chain oxidation products of astaxanthin, we screened for metabolites that matched possible reaction products of the short-chain aldehydes and ketones, and can be generated by endogenous plant enzymes. We discovered a relatively lowly abundant glycoside that exactly matched (by the mass of the molecular ion) a glucoside of 7-apoastaxanthinol (Figure 8C). In addition, a considerably more abundant glycoside, 9-apoastaxanthinol glucoside, was detected (Figure 8C). The annotation of these compounds was supported by in-source fragments characteristic of glucose conjugates and presence of the expected aglycone fragments (see Supplemental Dataset S1 for interpretation of the respective fragmentation patterns and molecular ions). The alcohol 9-apoastaxanthinol is derived from reduction of 9-apoastaxanthinone that is expected to be abundant, because it represents the complementary astaxanthin oxidation product to 10′-apoastaxanthinal. 9-Apoastaxanthinol glycoside is a plausible product of the action of endogenous enzyme activities present in tobacco cells. Tobacco is known to contain glycosides of several ionone-derived ionols, including the glycosides of 3-hydroxy-β-ionol, 3-hydroxy-7,8-dehydro-β-ionol, 3-oxo-α-ionol, and 3-hydroxy-5,6-epoxy-β-ionol (Cai et al., 2013). Specifically, 3-oxo-α-ionol, α-ionol, and β-ionol are reported substrates of UDP-sugar-dependent glycosyltransferases in *Nicotiana benthamiana*. Most of these enzymes have broad substrate specificity (Sun et al., 2019), and can be expected to have orthologs in *N. tabacum*. The abundances of the tentatively annotated 7- and 9-apoastaxanthinol glycosides and their isomers were independent of constitutive or inducible expression of the astaxanthin pathway (Figure 8C;Supplemental Dataset S1).

Substantially larger metabolic changes were detected in the polar metabolite fractions by LC-MS analysis. However, only few of these metabolites could be identified with the help of reference compounds. Notably, ascorbic acid and glutathione, two important antioxidants involved in detoxification of ROS and other free radicals were strongly depleted in the astaxanthin-synthesizing transplastomic plants. While the levels of both compounds were strongly reduced in the inducible lines, they were undetectable in the constitutive lines (Figure 8D).

In summary, while the constitutive *Nt*-AXT lines do not accumulate larger amounts of most of the direct enzymatic breakdown products of astaxanthin than the inducible *Nt*-iAXT plants, they appear to suffer from severe oxidative stress as evidenced by complete depletion of the key antioxidants ascorbate and glutathione.

## Discussion

In the course of this work, we generated transplastomic tobacco plants that express a synthetic astaxanthin biosynthetic operon under the control of a previously developed inducible expression system for plastids (Verhounig et al., 2010; Emadpour et al., 2015). The aim was to prevent the deleterious effects of constitutive expression of the astaxanthin pathway (Lu et al., 2017; Figure 2) by making operon expression dependent on application of the inducer metabolite theophylline.

Transplastomic expression of the astaxanthin operon under RAmpER control, indeed, largely alleviated the severe growth retardation entailed by constitutive operon expression (Figure 2). However, surprisingly, high-level astaxanthin synthesis did not require theophylline application (Figure 5; Supplemental Figure S1). In fact, astaxanthin levels in the uninduced *Nt*-iAXT plants were similar to those in *Nt*-AXT plants that express the operon constitutively (Figure 7). This observation, together with our finding that astaxanthin levels can be only moderately increased by RAmpER induction with theophylline (Figure 5), strongly suggests that astaxanthin accumulation is not limited by the expression strength of the operon. Instead, our data indicate that steps downstream of metabolite synthesis limit astaxanthin accumulation. As astaxanthin represents the end product of the pathway, this limitation likely lies in metabolite stability. It is well established that plastids do not have an unlimited storage capacity for lipophilic compounds such as carotenoids (Rabbani et al., 1998; Li et al., 2012; Kilambi et al., 2013; Nogueira et al., 2013), in that lipids and/or carotenoid-binding proteins are needed to safely sequester carotenoids and protect them from degradation. Therefore, based on the similar astaxanthin accumulation levels in *Nt*-AXT and *Nt*-iAXT plants (and the limited inducibility of the pathway), we reasoned that the astaxanthin levels attained in our transplastomic plants are close to the upper limit that can be reached without additional genetic interventions (see below). Consequently, any future engineering effort towards further increasing astaxanthin accumulation will likely need to target carotenoid turnover (e.g. by down-regulating carotenoid-cleaving enzymes) and/or the carotenoid sequestration capacity of the plastid (e.g. by enhancing lipid biosynthesis, overexpressing carotenoid-binding proteins, or stimulating plastoglobule biogenesis).

Our finding that astaxanthin accumulation levels (and accumulation of red pigment granules within chloroplasts; Figure 6) were very similar in *Nt*-AXT and *Nt*-iAXT plants, raised the question of what causes the strong growth phenotype of transplastomic plants that express the astaxanthin operon constitutively from the strong rRNA operon promoter (Lu et al., 2017; Figure 2). Based on the above considerations, it seems likely that massive turnover of astaxanthin in the constitutive plants results in energy depletion (due to a hyperactive futile cycle of synthesis and degradation) and/or depletion of precursors or intermediates of isoprenoid metabolism, thus impairing metabolic pathways that are connected to carotenoid biosynthesis. The latter hypothesis may gain circumstantial support from the strong reduction in chlorophyll accumulation seen in *Nt*-AXT plants (Figure 7). Overexpression of phytoene synthase in transgenic plants has revealed that enhanced geranylgeranyl pyrophosphate (GGPP) flux through carotenoid biosynthesis leads to insufficient GGPP substrate availability for chlorophyll biosynthesis (Camagna et al., 2019). Thus, a highly active astaxanthin pathway (that, in *Nt*-AXT plants, is further enhanced by a continuous turnover) likely depletes GGPP and reduces chlorophylls and gibberellins.

With the exception of α-citral (Figure 8, A–C; Supplemental Dataset S1), we did not see a strong over accumulation of known enzymatic degradation products of astaxanthin, when comparing the growth-retarded *Nt*-AXT plants with the inducible *Nt*-iAXT plants. However, it should be noted that not all products of astaxanthin catabolism are readily detectable by mass spectrometry. Also, we currently cannot exclude the possibility that some of the many unannotated polar compounds that accumulated differentially in *Nt*-AXT and *Nt*-iAXT plants (Supplemental Dataset S1) represent downstream degradation products of astaxanthin. Nonetheless, our data provide insight into astaxanthin catabolism in planta, in that several previously unknown degradation products were detected (Figure 8C;Supplemental Dataset S1). The observed accumulation of α-citral in *Nt*-AXT plants (Figure 8B) is particularly interesting in that this product of carotenoid breakdown acts as a strong microtubule-inhibiting cytotoxin in plants (Chaimovitsh et al., 2010), thus raising the possibility that its accumulation contributes to the serious growth phenotype of the constitutive plants.

The most striking difference in metabolism between constitutive and inducible lines was the virtually complete absence of ascorbic acid and glutathione from the constitutive plants (Figure 8D). This finding may suggest that the massive turnover of astaxanthin in the *Nt*-AXT plants leads to complete exhaustion of the antioxidative system. Ascorbic acid and glutathione play a crucial role in ROS detoxification and stress protection, both independently and jointly in the glutathione-ascorbate cycle (also known as Asada-Halliwell pathway; Gill and Tuteja, 2010; Vidal-Meireles et al., 2017; Hasanuzzaman et al., 2019). It thus seems likely that the transplastomic plants expressing the astaxanthin pathway constitutively suffer from strong (photo)oxidative damage, due to depletion of the key antioxidants ascorbate and glutathione.

In summary, transplastomic expression of the astaxanthin pathway under RAmpER control largely alleviates the severe growth retardation associated with constitutive pathway expression without causing a reduction in astaxanthin accumulation. Our data also suggest that oxidative stress resulting from a depleted antioxidative system is a major contributor to the mutant phenotype seen in the constitutive plants. Finally, the data obtained in the present study provide evidence for astaxanthin accumulation being limited by the sequestration capacity of the plastid. Therefore, in addition to isoprenoid precursor provision, carotenoid degradation, and carotenoid storage represent worthwhile targets of future engineering efforts towards optimizing the production of high-value carotenoids and related isoprenoids in plants. Genetic interventions that are worthwhile to test include overexpression of 1-deoxy-d-xylulose-5-phosphate synthase, the rate limiting enzyme of the MEP pathway (Simpson et al., 2016), silencing of specific CCDs involved in carotenoid degradation (Moreno et al., 2021), and expression of the ORANGE (OR) protein to enhance the carotenoid accumulation capacity of the plastid (Lu et al., 2006; Yazdani et al., 2019).

## Materials and methods

### Plant material and growth conditions

Aseptic tobacco (*N.* *tabacum* cv Petit Havana) plants for transformation experiments were raised on Murashige & Skoog (MS) medium (Murashige and Skoog, 1962) containing 3% (w/v) sucrose. The light intensity in the growth cabinet was 40 µE m^−2^ s^−1^ and the light regime was 16 h light at 24°C and 8 h dark at 22°C. Regenerated transplastomic plants were raised under identical conditions, then transferred to soil and grown under standard greenhouse conditions (average light intensity: 150 µE m^−2^ s^−1^).

### Construction of vectors for plastid transformation

To assemble a plastid transformation vector for RAmpER-mediated expression of the astaxanthin pathway, a previously constructed synthetic operon (Lu et al., 2017) was employed. Site-directed mutagenesis was used to remove undesired NcoI and SalI restriction sites within the synthetic astaxanthin operon, producing construct pSAA12. The riboswitch-inducible T7 RNA polymerase cassette has been described previously (Emadpour et al., 2015). To avoid unwanted homologous recombination between duplicated expression signals (Rogalski et al., 2006; Li et al., 2011), the *Nt* T*rps16* 3′ UTR downstream of the T7 RNA polymerase coding region (Emadpour et al., 2015) was replaced with the *Cr* T*rbcL* (Figure 1A), yielding construct pSAA13. Finally, the synthetic astaxanthin operon was excised from pSAA12 by restriction digestion with the enzymes NheI and NcoI, and inserted into pSAA13 (cut with NcoI and XbaI), generating plastid transformation construct piAXT (Figure 1A).

### Plastid transformation and selection of transplastomic lines

Particle gun-mediated (biolistic) transformation of tobacco plastids was performed according to published protocols (Svab et al., 1990; Svab and Maliga, 1993). Briefly, young leaves from tobacco plants raised under sterile conditions were harvested and bombarded with vector DNA-coated gold particles using the DuPont PDS-1,000/He biolistic gun (Bio-Rad, Munich, Germany). Bombarded leaves were then cut into pieces of ∼5 × 5 mm and placed onto MS-based plant regeneration medium containing 500 mg L^−1^ spectinomycin. Primary spectinomycin-resistant shoots (obtained after a selection period of 2–3 months) were subjected to two additional rounds of regeneration on spectinomycin-containing medium to select against untransformed copies of the highly polyploid plastid genome (Greiner et al., 2020) and isolate homoplasmic transplastomic lines (Maliga, 2004; Bock, 2015). Regenerated homoplasmic shoots were rooted and propagated on MS medium supplemented with 500 mg L^−1^ spectinomycin. Finally, rooted plantlets were transferred to soil and grown to maturity under standard greenhouse conditions.

### Seed assays

To test for maternal transgene inheritance and confirm the homoplasmic status of the transplastomic lines, T1 seeds obtained from transplastomic plants were surface-sterilized by treatment with sodium ethanol and hypochlorite, and sown on MS medium containing 500 mg L^−1^ spectinomycin. The absence of antibiotic-sensitive seedlings from the progeny and the uniform pigmentation phenotype of all seedlings ultimately confirmed homoplasmy (Figure 1C; Bock, 2001).

### Isolation and analysis of nucleic acids

Leaf tissue samples were snap-frozen in liquid nitrogen and used for extraction of nucleic acids. A cetyltrimethylammonium bromide-based method was employed for isolation of total cellular DNA (Doyle and Doyle, 1990). Total plant RNA was extracted with the NucleoSpin^®^ RNA Plant Kit (Macherey-Nagel, Düren, Germany) following the protocol of the supplier. For RFLP analysis, samples of 3 μg total DNA were digested with the restriction enzyme BglII, separated by electrophoresis in 1% (w/v) agarose gels, and transferred onto Hybond nylon membranes (GE Healthcare) by capillary blotting. For northern blot analyses, total cellular RNA was electrophoretically separated in 1% (w/v) denaturing agarose gels, and blotted onto Hybond nylon membranes (GE Healthcare). Gel-purified PCR products or restriction fragments obtained from digested vectors were used as probes for RFLP and northern blot analyses. The fragments were radiolabeled with [α-32P]dCTP by random priming using the Multiprime DNA labeling system (GE Healthcare). Hybridizations were performed at 65°C using standard protocols. Signals were analyzed with a Typhoon Trio+ variable mode imager (GE Healthcare). A *psaB*-specific hybridization probe was generated by PCR using primers pPsaB104 and pPsaB105, a *NpLyc*-specific probe was produced with primers pSAA124_NpLyc_1 and pSAA69_ast_seq, and a *T7RNAP*-specific probe was obtained with primers pSAA100_197seq6 and pSAA117_197seq2 (Supplemental Table S1). Hybridization probes for *BsCrtW and BsCrtZ* were generated by digesting vector piAXT with the restriction enzymes NdeI/BamHI (737 bp fragment) or BglII (309 bp fragment), respectively.

### Quantification of pigments

Chlorophylls and carotenoids were extracted from leaf tissue and quantified by chromatography as described previously (Agrawal et al., 2020).

### Sample preparation for microscopy

Light microscopy images were obtained with an Olympus Epi-Fluorescence Microscope (BX-51) essentially as described previously (Lu et al., 2017). Samples for TEM were prepared using published procedures (Lu et al., 2017; Armarego-Marriott et al., 2019) with minor modifications. Briefly, leaf samples were fixed in 2.5% (v/v) glutaraldehyde in 50 mM sodium cacodylate (pH 7.4) containing 5 mM CaCl_2_ for 1 h under vacuum. Fixation was continued at 4°C overnight, and followed by post-fixation with 1% (w/v) OsO_4_ and 0.8% (w/v) K_3_Fe(CN)_6_ in 50 mM cacodylate buffer (pH 7.4) for 2 h at 4°C. After rinsing the leaf samples, en bloc staining of the tissue was performed by incubation in 2% (w/v) aqueous uranyl acetate for 2 h at root temperature. Following dehydration in acetone, embedding in Epon-812 (Science Services GmbH, Munich, Germany) was carried out using standard protocols. For electron microscopy, ultrathin sections (50–70 nm) were cut with diamond knives, contrasted with 2% (w/v) uranyl acetate and lead citrate, and examined in a Zeiss EM 912 Omega transmission electron microscope (Carl Zeiss, Oberkochen, Germany).

### Analysis of volatile organic compounds

Volatile organic compound (VOC) emission from tobacco leaf material was measured by headspace solid phase micro extraction gas chromatography coupled to mass spectrometry (SPME-GC-MS) as described earlier (Agudelo-Romero et al., 2013; Agudelo-Romero et al., 2015; Fuentes et al., 2016). Briefly, leaves were harvested and snap-frozen in liquid nitrogen. After processing by a cryogenic grinding robot (Labman, North Yorkshire, UK), 500 ± 10 mg of frozen leaf powder was transferred to pre-cooled 20 mL head-space vials. Prior to SPME-GC-MS analysis, the samples were incubated for at least 1 h at 15°C in closed vials followed by an incubation at 50°C for 10 min. SPME-GC-MS analysis was done in a randomized block design of replicate material from the wild type (*n* = 3), Nt-*i*AXT (*n* = 6), and Nt-AXT plants (*n* = 3). The profiles were recorded by GC coupled to electron impact ionization/quadrupole MS using an Agilent 6890N24 gas chromatograph connected to an Agilent 5975B VL mass spectrometer (Agilent Technologies, Böblingen, Germany) fitted with a 60 m DB-624 capillary column of 0.25 mm internal diameter and 1.40 μm film thickness (Agilent Technologies Deutschland GmbH, Waldbronn, Germany), and with a StableFlex^TM^ SPME fiber coated with 65 µm polydimethylsiloxane/divinylbenzene (Supelco, Bellefonte, USA). Chromatography data processing of visually controlled data files and manually supervised metabolite identification were performed according to published procedures (Vallarino et al., 2018). Criteria for metabolite identification were the presence of at least three specific and selective mass fragments and, in the case of verification by authenticated reference substances, a retention time deviation <1.0%. VOC annotations were by mass-spectral match using the reference mass spectra of the National Institute of Standards and Technology mass spectral search and comparison program (NIST version 2.3) and the Golm Metabolome Database (Kopka et al., 2005). VOC annotation and confirmation by reference substances are reported in Supplemental Dataset S1.

Relative changes of compounds in VOC profiles from equal amounts of leaf tissue were analyzed based on mass-spectral abundance of specific and selective mass fragments after normalization to the sum of abundances of all recorded compounds, thus giving relative abundance values. Compounds of interest were selected by the following criteria: (1) presence in Nt-*i*AXT or Nt-AXT lines and absence from the wild type or vice versa, (2) complete pairwise comparisons using Student’s *t* tests, and (3) analysis of qualitative or quantitative differences between Nt-*i*AXT and Nt-AXT lines. Significance values of homoscedastic and heteroscedastic two-tailed t tests, and the sources of reference compounds are listed in Supplemental Dataset S1.

### Analysis of lipophilic and polar metabolites

Preparation of lipophilic and polar metabolite fractions was carried out by a methanol/methyl-tertiary-butyl-ether/water extraction method described previously (Giavalisco et al., 2011; Armarego-Marriott et al., 2019). Briefly, 100 mg (+/−10 mg) of frozen leaf powder were transferred to pre-cooled 2 mL round bottom micro-centrifuge vials (Eppendorf AG, Hamburg, Germany), and metabolites were extracted by adding 1 mL of precooled (−20°C) methyl-tertiary-butyl-ether: methanol mixture (3:1, v/v). Samples were incubated for 10 min at 4°C on an orbital shaker followed by 10 min in an ultrasonication bath. Subsequently, 500 µL water: methanol (3:1, v/v) were added and, after thorough mixing, the liquid phase was separated by a 5 min centrifugation at 16,000*g* in a micro-centrifuge at 4°C. The upper phase (700 µL) was transferred to a fresh tube and dried by speed vacuum concentration for analysis of the lipid fraction. The lower semi-polar phase (150 µL) was dried for analysis of the polar metabolite fraction. Dried fractions were stored at −80°C until further analysis.

The lipophilic fraction was analyzed by Ultra-Performance Liquid Chromatography (UPLC) separation using a Waters Acquity UPLC system (Waters, Eschborn, Germany), a C8 reversed-phase column of 100 mm × 2.1 mm with 1.7 μm particle size (Waters), and mobile phases of buffer A (water with 1% (v/v) 1 M NH_4_Ac and 0.1% (v/v) acetic acid) and buffer B (acetonitrile:isopropanol 7:3 v/v, containing 1% 1 M (v/v) NH_4_Ac, and 0.1% (v/v) acetic acid) according to published protocols (Pant et al., 2015). The polar fraction was analyzed by an HSST3 C18 reversed phase column of 100 mm × 2.1 mm with 1.8 µm particle size (Waters) and mobile phases of buffer A (0.1% (v/v) formic acid in H_2_O) and buffer B (0.1% (v/v) formic acid in acetonitrile) using published methods (Giavalisco et al., 2011).

LC-MS chromatogram data files were acquired by an Exactive^TM^ mass spectrometer (Thermo-Fisher, http://www.thermofisher.com) in full-scan mode using published settings (Giavalisco et al., 2011; Pant et al., 2015). Each lipophilic and polar fraction was analyzed twice, in both positive and negative ion mode. The GeneData software (https://www.genedata.com/) was used for chromatogram data pre-processing that included baseline correction, chemical noise subtraction, chromatogram alignment, peak detection, and de-isotoping of mass isotopologues. The resulting matrix of mass features contained: observed exact mass, exact mass range, m/z charge, observed retention time, and retention time range across all analyzed samples (Supplemental Dataset S1). GeneData processing suggested a molecular formula and an interpretation of molecular ion or adduct type. These suggestions were manually curated and in part verified by commercially available reference substances (Supplemental Dataset S1). The astaxanthin reference was ≥97% pure and isolated from *Blakeslea trispora* (Sigma-Aldrich, SML0982). The reference compound and the astaxanthin extracted from Nt-*i*AXT and Nt-AXT lines contained the same main isomer and had similar isomer patterns (Supplemental Dataset S1). Reference substances of astaxanthin metabolization products were not available, annotation criteria and relative abundance data are reported in Supplemental Dataset S1.

Relative changes of compounds in positive and negative mode analyses of the polar and lipid fractions from equal amounts of leaf tissue were analyzed after normalization of mass-spectral abundances of mass features to the sum of abundances of all recorded mass features, thus giving relative abundance values. Compounds of interest were selected as described for the VOC profiling.

## Accession numbers

Sequence data from this article can be found in the GenBank/EMBL data libraries under accession numbers_MF580999.1 (synthetic astaxanthin operon), M38308.1 (T7 RNA polymerase gene), and NC_001879.2 (*N. tabacum* plastid genome).

## Supplemental data

The following materials are available in the online version of this article.

**Supplemental Table S1.** List of synthetic oligonucleotides used as PCR primers in this study.

**Supplemental Figure S1.** Time course analysis of astaxanthin accumulation in leaf number 8 of plants at the 12-leaf stage.

**Supplemental Dataset S1.** Selected compounds from a comprehensive metabolomic screening for metabolites associated with inducible and/or constitutive astaxanthin synthesis.

## Supplementary Material

kiab428_Supplementary_Data

## Notes

S.A. performed the research; S.A., D.K., S.R., A.E., A.P.H., J.K., and R.B. designed the research and analyzed the data; S.A. and R.B. wrote the article with input from the other authors.

The author responsible for distribution of materials integral to the findings presented in this article in accordance with the policy described in the Instructions for Authors (https://academic.oup.com/plphys/pages/general-instructions) is: Ralph Bock (rbock@mpimp-golm.mpg.de).
