# Supplementary material for: Riboswitch-mediated inducible expression of an astaxanthin biosynthetic operon in plastids
Source: Plant Physiol. 2021 Sep 7;188(1):637–52. doi: 10.1093/plphys/kiab428 (PMC8774745; doi:10.1093/plphys/kiab428)
Supplement: kiab428_Supplementary_Data [file kiab428_supplementary_data.zip › kiab428-suppl_data/Bock_Suppl Mat_final.pdf]

SUPPLEMENTAL MATERIALS

SUPPLEMENTAL TABLES

Supplemental Table S1. List of synthetic oligonucleotides used as PCR primers in this study.

**Oligonucleotide**	**Sequence (5' → 3')**
pSAA124_NpLyc_1	ACTTTTCTTTATGCGATGCCCTT
pSAA69_ast_seq	ACAAGTATTTGAAGCATGAGAGAACAA
pSAA100_197seq6	CCTTTCGCCAGCGTAAGCAG
pSAA117_197seq2	TTACATGCCTGAGGTGTAC
pPsaB104	CCCAGAAAGAGGCTGGCCC
pPsaB105	CCCAAGGGGCGGGAACTGC

SUPPLEMENTAL FIGURES

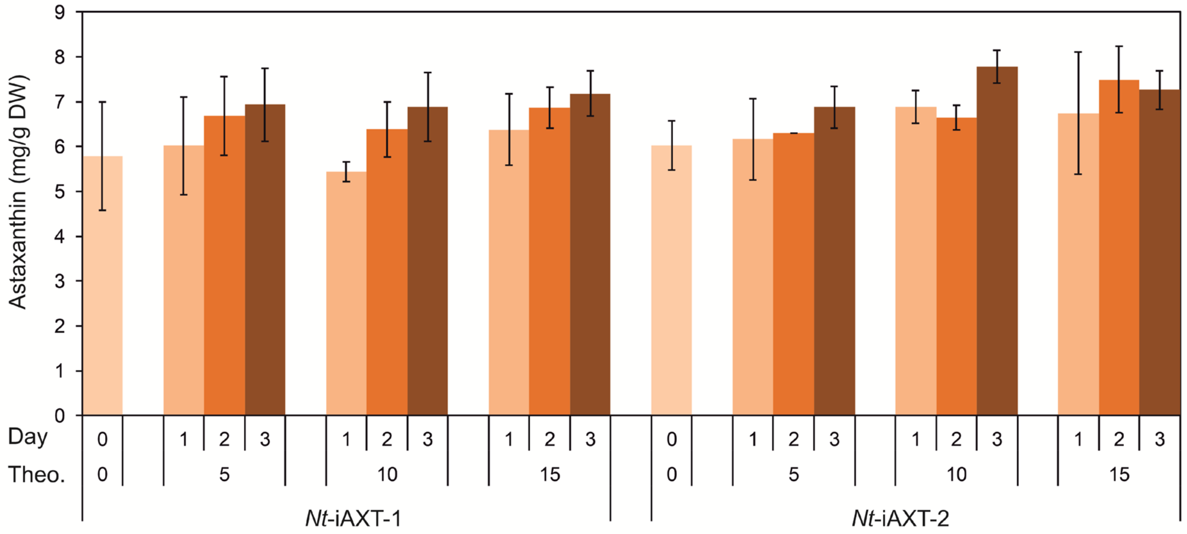

Supplemental Figure S1. Time course analysis of astaxanthin accumulation in leaf number 8 of plants at the 12-leaf stage. Riboswitch induction was performed with 5 mM, 10 mM or 15 mM theophylline (Theo.) for three days. Error bars indicate the SD (n=3).
